# Attitudes toward artificial intelligence tools in university students: associations with body appreciation and e-healthy diet literacy — implications for digital health education

**DOI:** 10.1186/s12909-026-09506-y

**Published:** 2026-05-22

**Authors:** Nida Nur Adiyan, Nezihe Otay Lule

**Affiliations:** 1 Department of Nutrition and Dietetics, Faculty of Health Sciences, Hasan Kalyoncu University, Gaziantep, Turkey; 2 Department of Nutrition and Dietetics, Faculty of Health Sciences, Gaziantep University, Gaziantep, Turkey

**Keywords:** Artificial intelligence, Attitudes toward artificial intelligence, Body appreciation, eHealth literacy, Healthy diet literacy, University students

## Abstract

**Background:**

This cross-sectional study aimed to determine attitudes toward the use of artificial intelligence tools, body appreciation, and e-healthy diet literacy (e-HDL) levels among university students, and to analytically evaluate the relationships among these variables.

**Methods:**

This cross-sectional study was conducted among 440 undergraduate university students aged 18–30 years enrolled at Gaziantep University. Data were collected with web-based questionnaire that included a Descriptive Information Form, the Artificial Intelligence Attitude Scale-Short Form (AIAS-4), the Body Appreciation Scale (BAS), and the e-HDL Scale.

**Results:**

The mean AIAS-4, BAS, and e-HDL total scores were 5.47 ± 2.62, 38.91 ± 9.00, and 37.89 ± 7.04, respectively. AIAS-4 scores were positively correlated with both BAS and e-HDL total scores. Male students had significantly higher AIAS-4 scores than female students, whereas female students had significantly higher BAS scores. Male students had significantly higher AIAS-4 scores than female students, whereas female students had significantly higher BAS scores; no significant sex difference was observed in e-HDL total scores. AIAS-4 and BAS scores differed significantly across BMI categories, while e-HDL total scores did not. In multiple linear regression analysis, higher BAS scores (β = 0.325, *p* < 0.001), higher e-HDL total scores (β = 0.247, *p* < 0.001), and higher BMI (β = 0.107, *p* = 0.021) were independently associated with higher AIAS-4 scores, whereas female sex was independently associated with lower AIAS-4 scores (β = -0.138, *p* = 0.003). Notably, BMI was not significantly correlated with AIAS-4 scores in the bivariate analysis (*r* = 0.083, *p* > 0.05), yet emerged as a significant independent predictor in the multivariable model (β = 0.107, *p* = 0.021). This discrepancy is consistent with a suppression effect, whereby sex — which is associated with both BMI and AI attitudes — masks the independent contribution of BMI in unadjusted analyses. After adjustment for sex and other covariates in the multivariable model, the association between BMI and AI attitudes became statistically detectable. This finding underscores the importance of multivariable adjustment when examining predictors that are inter-correlated. Age was not significantly associated with AIAS-4 scores. The regression model explained 22.0% of the variance in AIAS-4 scores.

**Conclusion:**

Among university students, more positive attitudes toward artificial intelligence tools were associated with higher body appreciation and e-HDL. In addition, sex and BMI were independently related to AI attitudes, whereas age and year of study were not. Although nearly all participants reported using AI, the regression model explained 22% of the variance in AI attitudes, indicating that the drivers of more positive attitudes remain multifaceted and warrant further investigation.

## Introduction

In this era of automation of assets and processes and the integration of digitalization into our lives, artificial intelligence (AI) has become an indispensable tool offering innovative solutions in many scientific fields, from engineering to medicine, pharmacy to nutrition [21, 31]. AI is attracting increasing attention owing to its capacity to analyze large datasets, recognize patterns, and generate personalized recommendations [5, 25]. In the context of this study, ‘AI tools’ refers broadly to AI-based applications that students may encounter in daily life, including generative AI systems such as large language model-based chatbots, as well as predictive applications used for dietary assessment or health information retrieval. The AIAS-4 scale used in this study captures general attitudes toward AI, reflecting this broad conceptualisation of AI engagement among young adults. Within this landscape, one of the most concrete reflections of this interest is in the field of nutritional science, where AI has promising applications in a wide variety of areas such as diet assessment, food recognition, personalized nutrition planning, and chronic disease management [17, 28]. These applications represent significant potential not only for increased efficiency and accuracy but also for access to quality nutritional advice, especially in resource-limited environments [25, 30]. In this digital transformation environment, it is noteworthy that young adults (18–30 years old) use AI tools and chatbots more than other age groups for various reasons, including limited budgets, social pressures, and academic concerns (52%) [9].

University life is a critical transitional period for establishing healthy eating habits, where individuals take full responsibility for their own dietary habits for the first time [29]. However, this period also brings with it the risk of developing unhealthy eating habits due to factors such as economic constraints, academic stress, lack of time, and lack of nutritional knowledge [13, 18]. These challenges can lead students to opt for energy-dense, low-nutrient options such as fast food, processed snacks, and sugary drinks [3, 10], which in turn lays the groundwork for problems such as overweight and obesity, which can be seen in over 30% of the university population [26, 27].

Another critical issue affecting university students is body dissatisfaction. Academic and social pressures, along with exposure to the perception of an “ideal body,” can lead to high rates of body dissatisfaction among students (63.5–71.7%) [16], a phenomenon particularly pronounced among female students [15]. Body dissatisfaction constitutes a significant risk factor for disordered eating behaviors such as anorexia, bulimia, and binge eating disorder (25-38.3%), profoundly impacting both the physical and emotional health of students [4, 23, 34].

In the face of these complex psychosocial and nutritional challenges, nutritional literacy, defined as individuals’ ability to acquire, understand, and apply healthy eating information, is gaining significant importance [19, 20]. A new dimension brought about by the digital age is e-healthy diet literacy, encompassing the ability to browse, understand, interpret, and apply nutritional information through electronic resources [12]. For young adults, who have adapted most rapidly to digitalization, this concept, which refers to the capacity to critically evaluate nutritional information from the internet and AI-based tools, has become key to making healthy eating decisions.

A plausible theoretical mechanism linking body appreciation to AI attitudes may involve self-efficacy and psychological openness. Individuals who hold more appreciative views of their bodies may also tend to approach novel technologies with greater confidence and lower anxiety, particularly when AI-generated content pertains to health or dietary behaviours. Conversely, individuals with lower body appreciation may be more likely to perceive AI feedback related to nutrition or body weight as threatening or judgmental, potentially fostering more ambivalent attitudes toward such tools. While this proposed mechanism remains speculative in the absence of direct empirical evidence, it offers a theoretically coherent basis for the observed association and warrants investigation in future studies. Taken together, these three constructs — AI attitudes, body appreciation, and e-healthy diet literacy — may form an interrelated framework in which psychosocial well-being and digital health competence jointly shape students’ engagement with AI-based health tools.

The literature has addressed AI applications in nutrition, university students’ nutritional problems, body appreciation, and nutritional literacy separately. However, the combined effect of e-healthy diet literacy (e-HDL) and body appreciation on attitudes toward nutrition-focused AI tools has not been sufficiently investigated. For example, a student with high e-HDL may critically evaluate AI suggestions, while a student with lower body image may see AI as supportive or as a potential pressure source. Examining whether e-HDL and body appreciation are associated with attitudes toward AI tools may help address this gap. Body appreciation was selected as the psychosocial variable of interest for several reasons. University students represent a population particularly vulnerable to body image concerns, which are prevalent in this age group and have been linked to disordered eating behaviours and health information-seeking patterns. Furthermore, given the nutritional focus of the study, individuals’ perceptions of their bodies may plausibly shape how they engage with AI-generated dietary content — a relationship that, to our knowledge, has not been previously examined. While constructs such as technology self-efficacy and technology acceptance represent important complementary frameworks, their associations with AI attitudes have been more extensively studied; the present study therefore addresses a distinct and underexplored psychosocial dimension.

The aim of this study is to determine the attitudes of university students towards the use of artificial intelligence tools, their body appreciation, and their e-HDL levels, and to analytically evaluate the relationships between these variables. The study hypothesizes that there is a significant relationship between university students’ attitudes toward artificial intelligence tools and their e-HDL scores (H1), and between AI attitudes and body appreciation scores (H2). A significant relationship between e-HDL scores and body appreciation scores is also hypothesised (H3). Finally, it is hypothesised that individual characteristics such as sex, year of study, and BMI are significantly associated with attitudes toward artificial intelligence, body appreciation, and e-HDL scores (H4).

## Methods

### Study design and population

This cross-sectional study was conducted among undergraduate university students aged 18–30 years who were enrolled at Gaziantep University. Data were collected between 3 January 2026 and 31 March 2026 using a web-based survey administered through Google Forms. The survey link was distributed online by the researchers via digital communication platforms commonly used by students, including Instagram and WhatsApp. A convenience sampling approach was used to recruit participants.

The primary outcome of the study was students’ attitudes toward the use of artificial intelligence. Explanatory variables included participants’ descriptive characteristics, e-HDL, and body appreciation.

### Inclusion and exclusion criteria

The inclusion criteria were as follows: being between 18 and 30 years of age, being enrolled as a student at Gaziantep University, and providing electronic informed consent by selecting the statement “I voluntarily agree to participate in this study” at the beginning of the online questionnaire.

Participants aged under 18 years or over 30 years, those who were not enrolled at Gaziantep University, those who did not provide informed consent, and those who submitted incomplete questionnaires were excluded from the study. Only participants who completed the questionnaire in full were included in the final sample.

### Sample size and power analysis

The minimum sample size was calculated using GPower software. Based on a priori power analysis for bivariate correlation (two-tailed test), assuming a medium effect size (*r* = 0.20), a type I error rate of α = 0.05, and a statistical power of 0.95, the minimum required sample size was determined to be 314 participants. The final sample of 440 participants also provides sufficient power for the multivariable regression analysis with five predictors, yielding an estimated power exceeding 0.95 for the detection of a medium effect size (f² = 0.15) at α = 0.05. To account for the possibility of missing or incomplete data, the target sample size was increased by 15% (*n* = 48), resulting in a planned total sample of 362 participants. Ultimately, a total of 440 students who completed the survey were included in the final analysis.

### Data collection

Data were collected using a web-based questionnaire consisting of the Descriptive Information Form, the Artificial Intelligence Attitude Scale-Short Form, the Body Appreciation Scale, and the e-HDL Scale. All scales were administered in Turkish.

### Descriptive information form

The Descriptive Information Form was prepared by the researchers and included questions on sex, age, body weight, height, year of study, artificial intelligence use status (yes/no), primary purpose for using artificial intelligence, use of artificial intelligence for disease symptom inquiries (yes/no), history of consultation with a nutrition professional (yes/no), source of healthy nutrition information, receiving nutrition/diet recommendations from artificial intelligence (yes/no), beliefs regarding the accuracy of information obtained from artificial intelligence (accurate/undecided/inaccurate), frequency of receiving nutrition recommendations from artificial intelligence, and whether participants consulted an expert about nutrition recommendations obtained from artificial intelligence. Body mass index (BMI) was calculated based on self-reported body weight and height, which participants reported using open-ended text fields (weight in kilograms; height in centimetres), using the formula weight (kg) / height (m²).

### Artificial Intelligence Attitude Scale-Short Form (AIAS-4)

Attitudes toward artificial intelligence were assessed using the Artificial Intelligence Attitude Scale-Short Form (AIAS-4), originally developed by Grassini. The scale is unidimensional and consists of four items. Each item is rated on a 10-point Likert scale ranging from 1 (“strongly disagree”) to 10 (“strongly agree”). The overall score is calculated as the mean of the four item scores, with higher scores indicating a more positive attitude toward and greater acceptance of artificial intelligence technology [14]. The Turkish adaptation of the scale was performed by Arslan et al., and the Cronbach’s alpha coefficient of the Turkish version was reported as 0.90 indicating excellent internal consistency [6].

### Body Appreciation Scale (BAS)

Body appreciation was assessed using the Body Appreciation Scale, originally developed by Tylka and Wood-Barcalow. The scale consisted of 10 items rated on a 5-point Likert scale ranging from 1 (“never”) to 5 (“always”). There were no reverse-coded items. Total scores ranged from 10 to 50, with higher scores indicating greater body appreciation [7]. The Turkish adaptation of the scale was conducted by Bakalım and Taşdelen-Karçkay, and the Cronbach’s alpha coefficient of the Turkish version was reported as 0.94 indicating excellent internal consistency [8].

### e-HDL scale

The e-Healthy Diet Literacy Questionnaire, originally developed by Duong et al., was used to assess individuals’ digital healthy nutrition literacy. The scale consisted of 15 items and 5 subdimensions: finding e-healthy diet information, understanding e-healthy diet information, appraising e-healthy diet information, applying e-healthy diet information, and digital healthy diet literacy. Higher scores indicate greater e-HDL. The maximum possible total score is 71. Duong et al., [33]. The Turkish adaptation of the scale was performed by Onbaşı and Türker, and the Cronbach’s alpha coefficient of the Turkish version was reported as 0.77 indicating acceptable internal consistency [24]. No additional pretesting of the Turkish version was conducted prior to data collection in the present study, as the scale had been previously validated in a Turkish sample.

### Ethics approval

This study was conducted in accordance with the Declaration of Helsinki and approved by the Gaziantep University Scientific Research and Publication Ethics Committee for Social and Human Sciences (No. 2026/48, Date: 02/01/2026). Before accessing the questionnaire, all participants provided electronic informed consent by selecting the statement indicating that they voluntarily agreed to participate in the study. The study followed the STROBE reporting guidelines.

### Statistical analysis

All statistical analyses were performed using IBM SPSS Statistics for Windows, version 24.0 (IBM Corp., Armonk, NY, USA). Continuous variables were summarized as mean ± standard deviation (SD), and categorical variables were presented as number and percentage. The distribution of continuous variables was evaluated using histograms, skewness and kurtosis values, and normality tests. Given the relatively large sample size, minor deviations from normality in formal tests were interpreted together with distributional characteristics, and variables showing approximately normal distributions were analyzed using parametric methods. Associations among age, BMI, AIAS-4 total score, BAS score, and e-HDL total score were examined using Pearson correlation analysis. Comparisons between two groups according to sex were performed using the independent-samples t-test.

Differences in AIAS-4, BAS, and e-HDL total scores across year-of-study groups and BMI categories were evaluated using one-way analysis of variance (ANOVA). Effect sizes for ANOVA were expressed as eta squared (η²). For BMI category comparisons, Games–Howell post-hoc tests were used for pairwise group comparisons. Internal consistency reliability was assessed using Cronbach’s alpha coefficients for the AIAS-4 (α = 0.949), BAS (α = 0.963), and e-HDL (α = 0.676; standardised α = 0.713) in the current sample, with values ≥ 0.70 considered acceptable. Given the multidimensional structure of the e-HDL, its total scale alpha was interpreted with caution. All scale items were administered in accordance with the validated Turkish versions, and no items were omitted or adapted. Given the exploratory nature of the bivariate analyses and the number of statistical comparisons performed, these results should be interpreted with caution regarding type I error inflation. Effect sizes are reported for all group comparisons, and the multivariable regression model is considered the primary analytical framework of this study.

To identify factors independently associated with attitudes toward artificial intelligence, a multiple linear regression analysis was performed with AIAS-4 total score as the dependent variable. Predictors were selected a priori based on the study hypotheses and theoretical considerations: BAS and e-HDL total scores were included as the primary psychosocial and digital literacy predictors, while age, sex, and BMI were included as demographic covariates with established relevance to AI attitudes and the primary predictors in the literature. Age, sex, BMI, BAS score, and e-HDL total score were entered as independent variables. Regression coefficients were reported as unstandardized coefficients (B), standard errors (SE), standardized beta coefficients (β), 95% confidence intervals (CI), and p-values. Multicollinearity was assessed using variance inflation factor (VIF) and tolerance values. Prior to interpreting the regression model, standard diagnostic checks were performed: residual normality was assessed using P-P plots and the Kolmogorov–Smirnov test, homoscedasticity was evaluated by visual inspection of residual-versus-fitted-value plots, and influential observations were identified using Cook’s distance. All assumptions were considered adequately met. A two-tailed p-value < 0.05 was considered statistically significant.

## Results

A total of 440 university students were included in the study. The general characteristics of the participants are presented in Table 1. The mean age was 21.20 ± 1.91 years, and 70.5% of the participants were female. Most participants were in their second year of study (57.0%), and the mean BMI was 22.85 ± 4.02 kg/m². Based on BMI categories, 62.3% had normal weight, 20.5% were overweight, 11.8% were underweight, and 5.5% were obese. Overall, 97.5% of the participants reported using AI. The most common purpose of AI use was education (58.6%), followed by general information seeking (30.5%), whereas health-related use (4.3%) and lifestyle coaching (4.1%) were less frequent.

In addition, 74.1% reported using AI for health problems or symptom inquiries, 23.2% had previously consulted a nutrition professional, and 45.5% reported receiving diet/nutrition recommendations from AI. Among those who received diet/nutrition recommendations from AI (*n* = 200), 43.5% believed these recommendations were accurate, 55.5% were undecided, and 1.0% considered them inaccurate. Most of these participants reported receiving such recommendations occasionally (52.0%), and 42.0% reported consulting an expert about AI-based recommendations. The mean AIAS-4 score was 5.47 ± 2.62, the mean BAS score was 38.91 ± 9.00, and the mean e-HDL total score was 37.89 ± 7.04. The mean e-HDL subscale scores were 7.02 ± 2.40 for finding e-healthy diet information, 9.23 ± 5.01 for understanding e-healthy diet information, 6.51 ± 2.37 for appraising e-healthy diet information, 4.64 ± 1.83 for applying e-healthy diet information, and 10.50 ± 2.65 for digital healthy diet literacy (Table 1).

**Table 1:** General characteristics of participants

Variable (*n* = 440)		*n* (%) or Mean ± SD
Age *(years)*		21.20 ± 1.91
Sex	Female	310 (70.5)
	Male	130 (29.5)
Year of study	1st year	117 (26.6)
	2nd year	251 (57.0)
	3rd year	50 (11.4)
	4th year	22 (5.0)
BMI *(kg/m²)*		22.85 ± 4.02
BMI category	Underweight	52 (11.8)
	Normal weight	274 (62.3)
	Overweight	90 (20.5)
	Obese	24 (5.5)
AI use status	Yes	429 (97.5)
Main purpose of AI use	Do not use	11 (2.5)
	Education	258 (58.6)
	Health	19 (4.3)
	Lifestyle coaching	18 (4.1)
	General information	134 (30.5)
Use of AI for health problems or symptom inquiries *(Yes)*		326 (74.1)
Consultation with a nutrition professional *(Yes)*		102 (23.2)
Receiving diet/nutrition recommendations from AI *(Yes)*		200 (45.5)
Belief in the accuracy of AI-based recommendations*	Believes they are accurate	87 (43.5)
	Undecided	111 (55.5)
	Believes they are inaccurate	2 (1.0)
Frequency of receiving nutrition recommendations from AI*	Almost every day	20 (10.0)
	1–2 times/week	36 (18.0)
	1–2 times/month	37 (18.5)
	Occasionally	104 (52.0)
	Never	3 (1.5)
Consulting an expert about AI-based recommendations*	Yes	84 (42.0)
	No	116 (58.0)
AIAS-4 score		5.47 ± 2.62
BAS score		38.91 ± 9.00
e-HDL total score		37.89 ± 7.04
Finding e-healthy diet information		7.02 ± 2.40
Understanding e-healthy diet information		9.23 ± 5.01
Appraising e-healthy diet information		6.51 ± 2.37
Applying e-healthy diet information		4.64 ± 1.83
Digital healthy diet literacy		10.50 ± 2.65

Data are presented as mean ± standard deviation or n (%). Percentages marked with an asterisk (*) were calculated among participants who reported receiving diet/nutrition recommendations from AI (*n* = 200). *BMI* Body mass index, *AIAS-4* Artificial Intelligence Attitude Scale-Short Form, *BAS* Body Appreciation Scale, *e-HDL* e-Healthy Diet Literacy

Pearson correlation analysis showed that AIAS-4 total scores were positively correlated with BAS scores (*r* = 0.356, *p* < 0.001) and e-HDL total scores (*r* = 0.326, *p* < 0.001). BAS scores were also positively correlated with e-HDL total scores (*r* = 0.265, *p* < 0.001). In contrast, AIAS-4 total scores were not significantly correlated with age or BMI. BAS scores showed a weak negative correlation with BMI (*r* = -0.196, *p* < 0.001), whereas no significant correlations were observed between e-HDL total scores and age or BMI (Table 2).

**Table 2:** Pearson correlations of AIAS-4, BAS, and e-HDL total scores with age and BMI

Variable (*n* = 440)	AIAS-4 total score	BAS score	e-HDL total score
Age (years)	0.037	-0.053	0.066
BMI (kg/m²)	0.083	-0.196**	0.017
BAS score	0.356**	—	0.265**
e-HDL total score	0.326**	0.265**	—

Values are Pearson correlation coefficients. *AIAS-4* Artificial Intelligence Attitude Scale-Short Form, *BAS* Body Appreciation Scale, *BMI* Body mass index, *e-HDL* e-Healthy Diet Literacy

Values in bold indicate statistical significance (** *p* < 0.001)

Sex-based comparisons showed that male students had significantly higher AIAS-4 scores than female students (5.93 ± 2.95 vs. 5.28 ± 2.45, *p* = 0.027). In contrast, female students had significantly higher BAS scores than male students (39.56 ± 8.67 vs. 37.38 ± 9.59, *p* = 0.026). No significant sex difference was observed in e-HDL total scores (38.16 ± 7.24 vs. 37.24 ± 6.53, *p* = 0.208) (Table 3).

**Table 3:** Comparison of AIAS-4, BAS, and e-HDL according to sex

Variable	Male (*n* = 130) Mean ± SD	Female (*n* = 310) Mean ± SD	*p*-value
AIAS-4 score	5.93 ± 2.95	5.28 ± 2.45	**0.027**
BAS score	37.38 ± 9.59	39.56 ± 8.67	**0.026**
e-HDL total score	37.24 ± 6.53	38.16 ± 7.24	0.208

*p*-values were obtained using independent-samples t-test. Welch correction was considered when the assumption of homogeneity of variances was violated. *AIAS-4* Artificial Intelligence Attitude Scale-Short Form, *BAS* Body Appreciation Scale, *e-HDL* e-Healthy Diet Literacy

Values in bold indicate statistical significance (*p* < 0.05)

One-way ANOVA showed no significant differences in AIAS-4, BAS, or e-HDL total scores across year-of-study groups (AIAS-4: F(3,436) = 1.907, *p* = 0.128; BAS: F(3,436) = 0.195, *p* = 0.900; e-HDL: F(3,436) = 0.557, *p* = 0.644) (Table 4).

**Table 4:** Comparison of AIAS-4, BAS, and e-HDL total scores according to year of study

Variable	F	η²	*p*-value
AIAS-4 total score	1.907	0.013	0.128
BAS score	0.195	0.001	0.900
e-HDL total score	0.557	0.004	0.644

*p*-values were obtained using one-way ANOVA. η², eta squared. *AIAS-4* Artificial Intelligence Attitude Scale-Short Form, *BAS* Body Appreciation Scale, *e-HDL* e-Healthy Diet Literacy

One-way ANOVA revealed significant differences in AIAS-4 and BAS scores across BMI categories (AIAS-4: F(3,436) = 2.717, *p* = 0.044; BAS: F(3,436) = 3.487, *p* = 0.016), whereas e-HDL total scores did not differ significantly (F(3,436) = 1.014, *p* = 0.386). Post-hoc comparisons showed that participants with obesity had significantly higher AIAS-4 scores than those with normal weight (6.85 ± 2.58 vs. 5.31 ± 2.60, *p* = 0.042). In addition, participants with normal weight had significantly higher BAS scores than overweight participants (39.73 ± 8.75 vs. 36.90 ± 8.95, *p* = 0.048) (Table 5). However, it should be noted that the obese subgroup comprised only 24 participants, which limits the precision and stability of estimates for this category; these findings should therefore be interpreted with caution.

**Table 5:** Comparison of AIAS-4, BAS, and e-HDL total scores according to BMI category

Variable	Underweight (*n* = 52) Mean ± SD	Normal weight (*n* = 274) Mean ± SD	Overweight (*n* = 90) Mean ± SD	Obese (*n* = 24) Mean ± SD	*p*-value
AIAS-4 score	5.42 ± 2.59^**ab**^	5.31 ± 2.60^**a**^	5.62 ± 2.64^**ab**^	6.85 ± 2.58^**b**^	**0.044**
BAS score	39.63 ± 9.54^**ab**^	39.73 ± 8.75^**a**^	36.90 ± 8.95^**b**^	35.63 ± 9.35^**ab**^	**0.016**
e-HDL total score	39.19 ± 8.81	37.49 ± 6.95	38.16 ± 5.96	38.63 ± 7.51	0.386

*p*-values were obtained using one-way ANOVA. Post-hoc pairwise comparisons were performed using Games–Howell tests ^ab^. Significant pairwise differences were observed between obese and normal-weight participants for AIAS-4 scores and between normal-weight and overweight participants for BAS scores. Different superscript letters (a, b) indicate significant differences between groups based on Games–Howell post-hoc test, *p* < 0.05)

Values in bold indicate statistically significant results (*p* < 0.05)

In multiple linear regression analysis, the model including age, sex, BMI, BAS score, and e-HDL total score was statistically significant (F(5,434) = 24.494, *p* < 0.001) and explained 22.0% of the variance in AIAS-4 scores (R² = 0.220; adjusted R² = 0.211). Higher BAS scores (B = 0.095, β = 0.325, *p* < 0.001), higher e-HDL total scores (B = 0.092, β = 0.247, *p* < 0.001), and higher BMI (B = 0.069, β = 0.107, *p* = 0.021) were independently associated with higher AIAS-4 scores. Sex was also significantly associated with AIAS-4 scores (B = -0.791, β = -0.138, *p* = 0.003); given that sex was coded as 1 = male and 2 = female, the negative coefficient indicates that female students had lower AIAS-4 scores than male students. Age was not significantly associated with AIAS-4 scores (*p* = 0.624). Multicollinearity was not a concern, with VIF values ranging from 1.091 to 1.180 and tolerance values ranging from 0.847 to 0.916 (Table 6). Regression diagnostic checks confirmed that residuals were approximately normally distributed, no substantial heteroscedasticity was identified, and no influential observations were detected based on Cook’s distance.

**Table 6:** Multiple linear regression analysis for factors associated with AIAS-4 scores

Variable	B	SE	Standardized β	95% CI	*p*-value
Age	-0.030	0.061	-0.022	-0.150 to 0.090	0.624
Sex	-0.791	0.261	**-0.138**	-1.304 to -0.278	**0.003**
BMI	0.069	0.030	**0.107**	0.010 to 0.128	**0.021**
BAS score	0.095	0.013	**0.325**	0.069 to 0.121	**< 0.001**
e-HDL total score	0.092	0.016	**0.247**	0.060 to 0.124	**< 0.001**

Model statistics: R² = 0.220; adjusted R² = 0.211; F(5,434) = 24.494; *p* < 0.001

*B* unstandardized regression coefficient, *SE* Standard error, *β* Standardized regression coefficient, *CI* Confidence interval, *BMI* Body mass index, *BAS* Body Appreciation Scale, *e-HDL* e-Healthy Diet Literacy, *AIAS-4* Artificial Intelligence Attitude Scale-Short Form. Sex was coded as 1 = male and 2 = female. Dependent variable: AIAS-4 total score. All VIF<1.2

Bold values indicate statistical significance (*p* < 0.05)

## Discussion

In this study, more favorable attitudes toward artificial intelligence tools among university students were consistently linked to higher body appreciation and greater e-HDL, both at the correlational level and after adjustment in multivariable analysis. Although these correlations were statistically significant, they were small to moderate in magnitude (*r* = 0.265–0.356), indicating that the associations, while consistent and meaningful at the population level, have limited explanatory power at the individual level and should be interpreted with appropriate caution regarding their practical implications. Notably, body appreciation emerged as the strongest independent correlate of AI attitudes, followed by e-HDL, suggesting that students who perceived their bodies more positively and who were more competent in accessing and interpreting digital nutrition information also tended to report more positive attitudes toward AI tools. In addition, AI attitudes differed by sex and BMI, with male students and those with higher BMI showing higher AIAS-4 scores, whereas year of study was not associated with AI attitudes, body appreciation, or e-HDL. Taken together, these findings suggest that attitudes toward AI in this population may be shaped less by academic seniority and more by individual psychosocial and digital health-related characteristics.

Nearly all participants reported using AI, and nearly half received nutrition-related recommendations. This high level of AI engagement is consistent with broader trends in digital technology use among young adults, suggesting that AI represents an increasingly accessible — though not universally trusted — source of health-related information in this population. Blahosova et al., [9]. Notably, almost half of the participants reported receiving dietary recommendations from AI, while a considerable proportion did not seek professional advice regarding these recommendations. This pattern may raise concerns about the potential for misinformation or misinterpretation, particularly in the context of nutrition, where individualized and evidence-based guidance is essential [22, 24]. This is particularly concerning given the well-documented risk of factual inaccuracies in AI-generated content — commonly referred to as ‘hallucinations’ — which may be especially consequential in nutrition and health contexts where misinformation can directly affect dietary behaviour and health outcomes [5]. The observation that more than half of students who received AI-based dietary recommendations remained uncertain about their accuracy further underscores the need for educational interventions that develop students’ capacity to critically evaluate AI-generated health information. At the same time, it highlights the growing reliance on AI as an alternative or complementary source of health information [35]. In this context, individuals’ ability to critically evaluate and effectively use such information is becoming increasingly important, highlighting an association between e-HDL and how AI-generated recommendations are perceived and utilized.

The positive correlation was observed between AIAS-4 scores and e-HDL levels, indicating that students with higher e-HDL tend to have more favorable attitudes toward artificial intelligence. Similarly, Alam et al., [2] reported that higher levels of digital health literacy were associated with more positive attitudes toward AI use among university students. One possible explanation for this relationship is that individuals with higher e-HDL may be better equipped to critically evaluate AI-generated information, which may be reflected in greater confidence when engaging with these tools. This capacity for critical appraisal may reduce uncertainty and increase trust in AI-based recommendations, thereby contributing to more positive attitudes. In addition, previous research has suggested that AI-supported applications may themselves contribute to the development of health literacy and facilitate more effective use of digital tools in decision-making processes [1]. Taken together, these findings point to a potentially bidirectional relationship between e-HDL and AI use, in which higher literacy enhances engagement with AI, while AI tools may also support the development of literacy-related skills [1]. However, not all studies have reported positive associations between digital health literacy and AI attitudes; some evidence suggests that individuals with higher health literacy may also express greater scepticism toward AI-generated recommendations, recognising their limitations more acutely [1]. This underscores the complexity of the relationship and the need for more nuanced investigation. A similar bidirectional relationship may exist between body appreciation and AI attitudes: while individuals with higher body appreciation may approach AI tools more openly, repeated positive experiences with AI-generated health content could also reinforce favourable body perceptions over time. Furthermore, it is equally plausible that students who already use AI frequently develop more positive attitudes through familiarity and repeated exposure — a mechanism consistent with the mere exposure effect — rather than entering AI engagement with pre-existing favourable attitudes. These alternative interpretations highlight the limitations of cross-sectional data in establishing the directionality of the observed associations, and underscore the need for longitudinal designs in future research.

Mean body appreciation was above the scale midpoint, with a significant positive association with AI attitudes. Students with higher body appreciation may perceive AI recommendations as supportive rather than threatening. Females had higher body appreciation than males, consistent with sociocultural pressures [11, 32]. The finding that male students reported higher AI attitudes despite lower body appreciation scores may reflect broader patterns of gender socialisation in relation to technology. Research consistently suggests that males report higher levels of technology self-efficacy and more positive attitudes toward digital tools, which may be attributable to differential exposure to technology-related activities during childhood and adolescence, as well as persistent gender norms associating technological competence with masculinity [2]. These sociocultural factors likely operate independently of body image, which may explain the divergent patterns observed for AI attitudes and body appreciation across sexes. BMI was negatively associated with body appreciation, while participants with higher BMI (especially obesity) had more favorable AI attitudes. Given the small size of the obese subgroup (*n* = 24), this finding should be regarded as exploratory and interpreted with caution pending replication in larger and more balanced samples. This suggests that individuals with higher BMI and lower body appreciation may turn to AI as accessible, non-judgmental guidance for eating behaviors and physical appearance. This interpretation is consistent with evidence suggesting that weight stigma and fear of judgment from healthcare professionals represent significant barriers to help-seeking among individuals with higher BMI [4]. In this context, AI tools may be perceived as offering a lower-stigma environment for accessing dietary guidance, free from the social evaluation associated with in-person professional consultations. Supporting this, previous research found that positive AI attitudes are associated with fewer disordered eating symptoms [6].

Multivariable analysis showed that body appreciation and e-HDL remained independently associated with AI attitudes, indicating distinct psychosocial and cognitive pathways. However, the model explained only 21.1% of the variance in AI attitudes (adjusted R² = 0.211), indicating that a substantial proportion of the variance (approximately 79%) remains unexplained by the variables examined in this study. Additional psychosocial, technological, and contextual factors — including prior AI experience, trust in technology, digital literacy beyond the nutritional domain, personality characteristics, and social influences — likely contribute meaningfully to AI attitudes in this population and should be examined in future longitudinal research [1, 5, 17, 21, 25, 28, 31]. Future studies may also benefit from examining the independent contributions of e-HDL subdimensions — particularly the critical appraisal subscale — to AI attitudes, rather than relying solely on the total score. Beyond the variables examined in this study, several broader theoretical considerations may help contextualise the findings. Attitudes toward AI tools are likely shaped not only by individual psychosocial characteristics but also by the perceived relational role of AI — for instance, whether it is viewed as a supportive guide or an impersonal information source — and this perception may interact with factors such as body appreciation or digital literacy in ways that future research should explore. Furthermore, trust-related concerns including privacy, transparency, and data security represent potentially important determinants of user acceptance in health technology contexts that were not directly assessed here. From a human-centric perspective, psychosocial factors such as body appreciation may not merely correlate with AI attitudes but could act as moderating influences on how individuals engage with digital health tools. Finally, cultural context — including local digital infrastructure and norms around health information-seeking — may shape AI attitudes in ways specific to the Turkish university setting, and future cross-cultural studies are needed to establish the broader generalizability of these findings. Given the cross-sectional design of this study, all observed associations are exploratory in nature and do not permit causal or directional inference.

### Implications for digital health education

The findings of this study carry several implications for digital health education. First, the positive association between e-healthy diet literacy and AI attitudes suggests that students who are better equipped to navigate and critically evaluate digital health information tend to approach AI tools more favourably. This underscores the value of integrating digital health literacy — including critical appraisal of AI-generated content — into university-level health education programmes. Second, the association between body appreciation and AI attitudes highlights the relevance of psychosocial well-being to students’ engagement with digital health technologies. Educational interventions that support positive body image alongside digital competence may foster more balanced and critical use of AI in health-related contexts. Third, these findings underscore the need for curriculum content that equips students with the skills to critically evaluate AI-generated health information and to seek professional guidance where appropriate — a competency of growing importance in health professions education. Finally, the sex-based differences observed in AI attitudes suggest that gender-sensitive approaches to AI literacy education may be warranted.

### Limitations

This study has several limitations. First, its cross-sectional design precludes any causal interpretation of the observed associations between attitudes toward artificial intelligence tools, body appreciation, and e-HDL. Second, the study was conducted at a single public university using an online, self-administered questionnaire distributed via social media platforms, which limits the generalizability of the findings to other age groups, institutions, or sociocultural settings. Furthermore, this recruitment approach introduces digital access bias, as students without regular internet access or those less engaged with digital platforms may have been systematically underrepresented. The entirely voluntary nature of participation also raises the possibility of volunteer bias, whereby students with stronger interest in AI or health-related topics may have been more inclined to respond, potentially inflating the prevalence of favorable AI attitudes in the sample. Additionally, field of study was not recorded, precluding analysis of whether academic discipline — particularly health sciences versus non-health disciplines — confounds or moderates the observed associations; this should be addressed in future studies. Additionally, the sample was predominantly female (70.5%) and consisted largely of second-year students (57.0%), which may limit the representativeness of the findings across sexes and academic years. Third, all data were based on self-report, which may have introduced recall bias, social desirability bias, and common method variance. In particular, body weight and height were self-reported, and BMI was therefore calculated indirectly rather than based on objective anthropometric measurements. Self-reported weight and height are subject to systematic misreporting, with a general tendency toward underestimation of body weight and overestimation of height, which may have resulted in misclassification of BMI categories — a concern that is particularly relevant for the obese subgroup, which was relatively small in the present sample. Furthermore, as all constructs were measured using self-administered questionnaires administered within the same survey instrument, common method variance cannot be excluded and may have artifactually inflated the magnitude of the observed correlations among AIAS-4, BAS, and e-HDL scores. In addition, although the AIAS-4 provides a practical brief assessment of general attitudes toward artificial intelligence, it may not fully capture domain-specific attitudes, trust, or actual patterns of AI use in health- and nutrition-related contexts. Furthermore, formal pilot testing of the questionnaire was not conducted prior to data collection, which represents an additional methodological limitation. Finally, some subgroup categories were relatively small, which may have reduced the stability of certain between-group comparisons.

This study also has important strengths. It examined attitudes toward artificial intelligence tools together with body appreciation and e-HDL within the same analytical framework, thereby addressing an underexplored area in university students. The relatively large sample size, use of validated measurement tools, and inclusion of multivariable regression analyses enhanced the robustness of the findings. These strengths provide a valuable foundation for future studies exploring how psychosocial and digital health-related characteristics are linked to AI attitudes in young adults.

## Conclusion

This study demonstrated that university students’ attitudes toward artificial intelligence tools are positively associated with body appreciation and e-HDL, while sex and body mass index also emerge as independent correlates. The prominent role of body appreciation suggests that individuals with more positive body perceptions may adopt a more open and confident approach toward AI-based health and nutrition applications. In addition, the independent association between AI attitudes and e-HDL highlights the importance of digital competencies in shaping how students engage with and benefit from AI-driven information. These findings suggest that educational programmes targeting both positive body image and digital health literacy — including critical appraisal of AI-generated nutrition information — may promote more effective, discerning, and responsible engagement with AI tools among university students.

## Data Availability

The datasets generated and/or analyzed during the current study are not publicly available due to institutional data protection policies but are available from the corresponding author on reasonable request.

## Abbreviations

AI: Artificial Intelligence
AIAS-4: Artificial Intelligence Attitude Scale-Short Form
BAS: Body Appreciation Scale
BMI: Body Mass Index
CI: Confidence Interval
e-HDL: e-Healthy Diet Literacy
SD: Standard Deviation
SE: Standard Error
